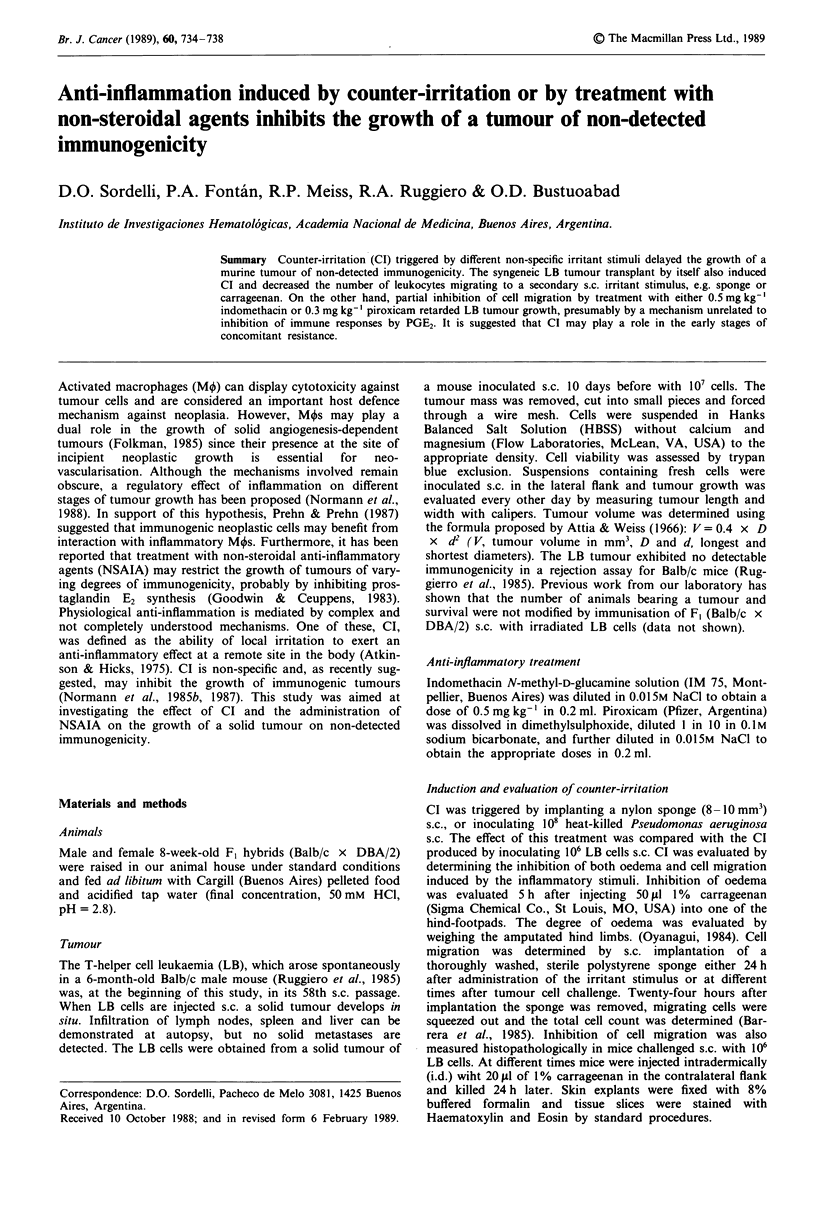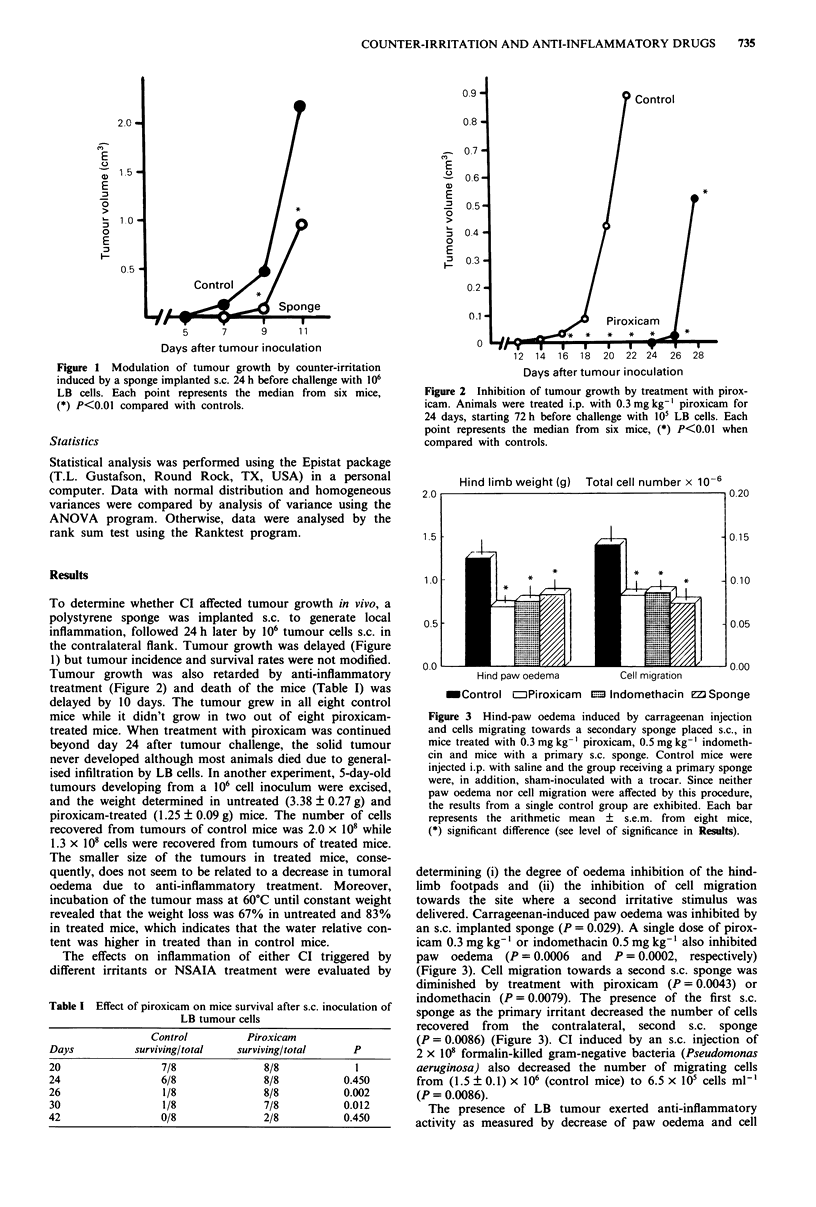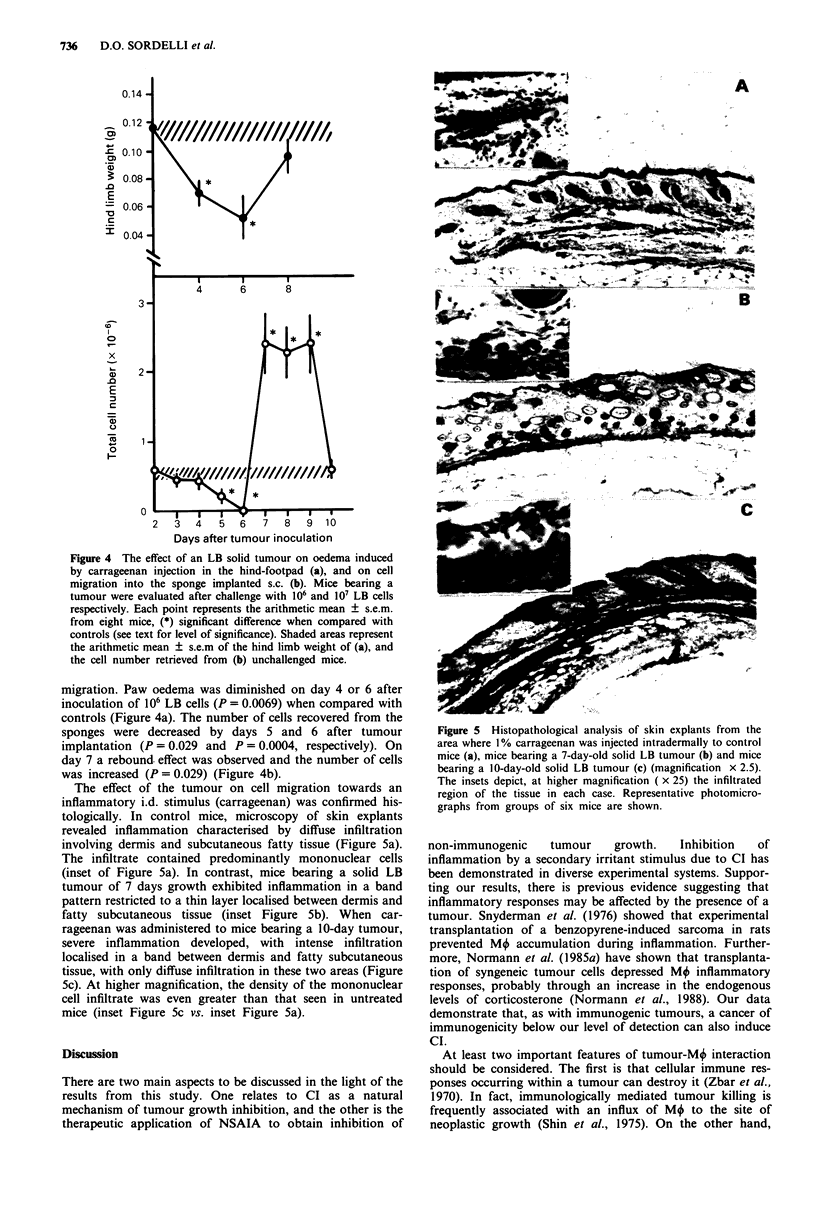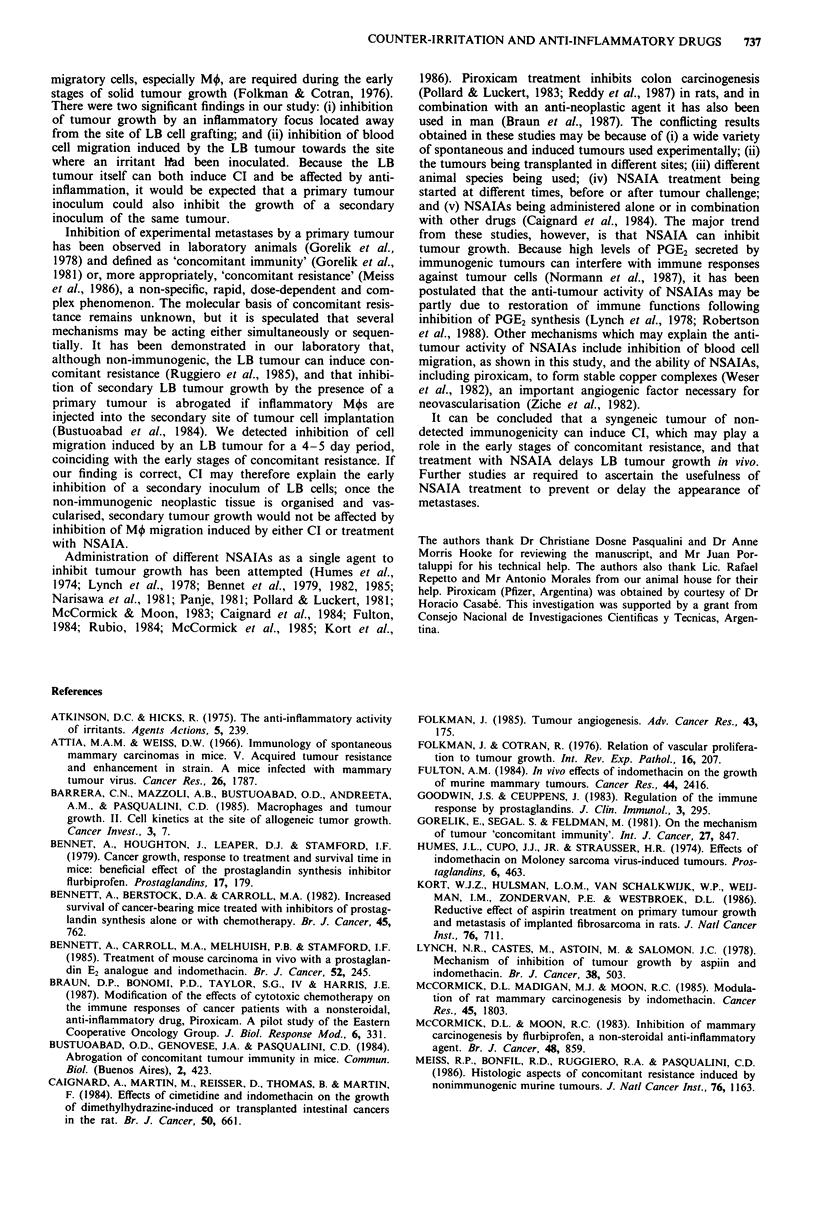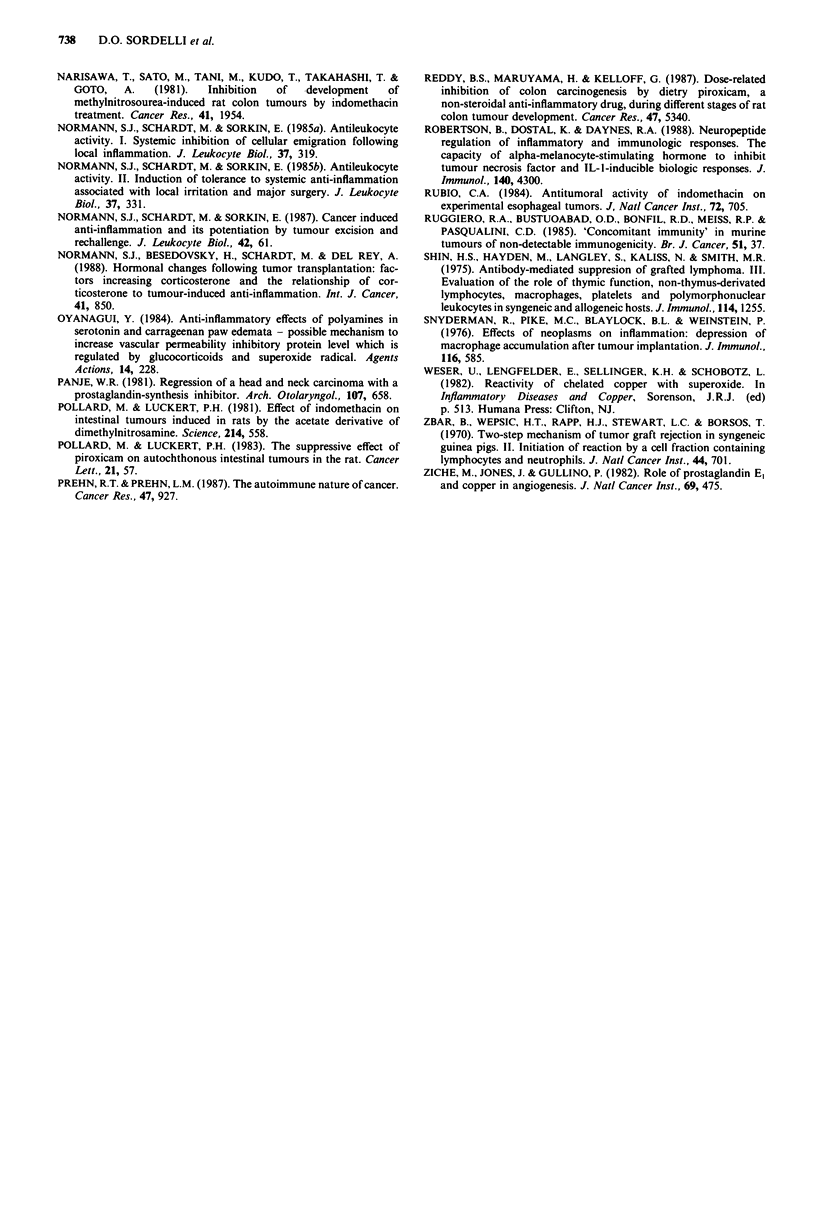# Anti-inflammation induced by counter-irritation or by treatment with non-steroidal agents inhibits the growth of a tumour of non-detected immunogenicity.

**DOI:** 10.1038/bjc.1989.349

**Published:** 1989-11

**Authors:** D. O. Sordelli, P. A. FontÃ¡n, R. P. Meiss, R. A. Ruggiero, O. D. Bustuoabad

**Affiliations:** Instituto de Investigaciones HematolÃ³gicas, Academia Nacional de Medicina, Buenos Aires, Argentina.

## Abstract

**Images:**


*Br. J. Cancer* (1989), **60**, 734–738 © The Macmillan Press Ltd., 1989

### Anti-inflammation induced by counter-irritation or by treatment with non-steroidal agents inhibits the growth of a tumour of non-detected immunogenicity

D.O. Sordelli, P.A. Fontán, R.P. Meiss, R.A. Ruggiero & O.D. Bustuoabad

*Instituto de Investigaciones Hematológicas, Academia Nacional de Medicina, Buenos Aires, Argentina.*

**Summary** Counter-irritation (CI) triggered by different non-specific irritant stimuli delayed the growth of a murine tumour of non-detected immunogenicity. The syngeneic LB tumour transplant by itself also induced CI and decreased the number of leukocytes migrating to a secondary s.c. irritant stimulus, e.g. sponge or carrageenan. On the other hand, partial inhibition of cell migration by treatment with either $0.5\,mg\,kg^{-1}$ indomethacin or $0.3\,mg\,kg^{-1}$ piroxicam retarded LB tumour growth, presumably by a mechanism unrelated to inhibition of immune responses by $PGE_2$. It is suggested that CI may play a role in the early stages of concomitant resistance.

Activated macrophages (M$\phi$) can display cytotoxicity against tumour cells and are considered an important host defence mechanism against neoplasia. However, M$\phi$s may play a dual role in the growth of solid angiogenesis-dependent tumours (Folkman, 1985) since their presence at the site of incipient neoplastic growth is essential for neovascularisation. Although the mechanisms involved remain obscure, a regulatory effect of inflammation on different stages of tumour growth has been proposed (Normann *et al.*, 1988). In support of this hypothesis, Prehn & Prehn (1987) suggested that immunogenic neoplastic cells may benefit from interaction with inflammatory M$\phi$s. Furthermore, it has been reported that treatment with non-steroidal anti-inflammatory agents (NSAIA) may restrict the growth of tumours of varying degrees of immunogenicity, probably by inhibiting prostaglandin $E_2$ synthesis (Goodwin & Ceuppens, 1983). Physiological anti-inflammation is mediated by complex and not completely understood mechanisms. One of these, CI, was defined as the ability of local irritation to exert an anti-inflammatory effect at a remote site in the body (Atkinson & Hicks, 1975). CI is non-specific and, as recently suggested, may inhibit the growth of immunogenic tumours (Normann *et al.*, 1985*b*, 1987). This study was aimed at investigating the effect of CI and the administration of NSAIA on the growth of a solid tumour on non-detected immunogenicity.

### Materials and methods

### *Animals*

Male and female 8-week-old $F_1$ hybrids (Balb/c × DBA/2) were raised in our animal house under standard conditions and fed *ad libitum* with Cargill (Buenos Aires) pelleted food and acidified tap water (final concentration, 50 mM HCl, pH = 2.8).

### *Tumour*

The T-helper cell leukaemia (LB), which arose spontaneously in a 6-month-old Balb/c male mouse (Ruggiero *et al.*, 1985) was, at the beginning of this study, in its 58th s.c. passage. When LB cells are injected s.c. a solid tumour develops *in situ*. Infiltration of lymph nodes, spleen and liver can be demonstrated at autopsy, but no solid metastases are detected. The LB cells were obtained from a solid tumour of a mouse inoculated s.c. 10 days before with $10^7$ cells. The tumour mass was removed, cut into small pieces and forced through a wire mesh. Cells were suspended in Hanks Balanced Salt Solution (HBSS) without calcium and magnesium (Flow Laboratories, McLean, VA, USA) to the appropriate density. Cell viability was assessed by trypan blue exclusion. Suspensions containing fresh cells were inoculated s.c. in the lateral flank and tumour growth was evaluated every other day by measuring tumour length and width with calipers. Tumour volume was determined using the formula proposed by Attia & Weiss (1966): $V = 0.4 \times D \times d^2$ ($V$, tumour volume in $mm^3$, $D$ and $d$, longest and shortest diameters). The LB tumour exhibited no detectable immunogenicity in a rejection assay for Balb/c mice (Euggierro *et al.*, 1985). Previous work from our laboratory has shown that the number of animals bearing a tumour and survival were not modified by immunisation of $F_1$ (Balb/c × DBA/2) s.c. with irradiated LB cells (data not shown).

### *Anti-inflammatory treatment*

Indomethacin *N*-methyl-D-glucamine solution (IM 75, Montpellier, Buenos Aires) was diluted in 0.015M NaCl to obtain a dose of $0.5\,mg\,kg^{-1}$ in 0.2 ml. Piroxicam (Pfizer, Argentina) was dissolved in dimethylsulphoxide, diluted 1 in 10 in 0.1M sodium bicarbonate, and further diluted in 0.015M NaCl to obtain the appropriate doses in 0.2 ml.

### *Induction and evaluation of counter-irritation*

CI was triggered by implanting a nylon sponge (8–10 $mm^3$) s.c., or inoculating $10^8$ heat-killed *Pseudomonas aeruginosa* s.c. The effect of this treatment was compared with the CI produced by inoculating $10^6$ LB cells s.c. CI was evaluated by determining the inhibition of both oedema and cell migration induced by the inflammatory stimuli. Inhibition of oedema was evaluated 5 h after injecting 50 µl 1% carrageenan (Sigma Chemical Co., St Louis, MO, USA) into one of the hind-footpads. The degree of oedema was evaluated by weighing the amputated hind limbs. (Oyanagui, 1984). Cell migration was determined by s.c. implantation of a thoroughly washed, sterile polystyrene sponge either 24 h after administration of the irritant stimulus or at different times after tumour cell challenge. Twenty-four hours after implantation the sponge was removed, migrating cells were squeezed out and the total cell count was determined (Barrera *et al.*, 1985). Inhibition of cell migration was also measured histopathologically in mice challenged s.c. with $10^6$ LB cells. At different times mice were injected intradermically (i.d.) wiht 20 µl of 1% carrageenan in the contralateral flank and killed 24 h later. Skin explants were fixed with 8% buffered formalin and tissue slices were stained with Haematoxylin and Eosin by standard procedures.

Correspondence: D.O. Sordelli, Pacheco de Melo 3081, 1425 Buenos Aires, Argentina.
Received 10 October 1988; and in revised form 6 February 1989.

**Figure 1** Modulation of tumour growth by counter-irritation induced by a sponge implanted s.c. 24 h before challenge with $10^6$ LB cells. Each point represents the median from six mice, (*) $P<0.01$ compared with controls.

**Figure 2** Inhibition of tumour growth by treatment with piroxicam. Animals were treated i.p. with 0.3 mg $kg^{-1}$ piroxicam for 24 days, starting 72 h before challenge with $10^5$ LB cells. Each point represents the median from six mice, (*) $P<0.01$ when compared with controls.

### *Statistics*

Statistical analysis was performed using the Epistat package (T.L. Gustafson, Round Rock, TX, USA) in a personal computer. Data with normal distribution and homogeneous variances were compared by analysis of variance using the ANOVA program. Otherwise, data were analysed by the rank sum test using the Ranktest program.

### Results

To determine whether CI affected tumour growth *in vivo*, a polystyrene sponge was implanted s.c. to generate local inflammation, followed 24 h later by $10^6$ tumour cells s.c. in the contralateral flank. Tumour growth was delayed (Figure 1) but tumour incidence and survival rates were not modified. Tumour growth was also retarded by anti-inflammatory treatment (Figure 2) and death of the mice (Table I) was delayed by 10 days. The tumour grew in all eight control mice while it didn't grow in two out of eight piroxicam-treated mice. When treatment with piroxicam was continued beyond day 24 after tumour challenge, the solid tumour never developed although most animals died due to generalised infiltration by LB cells. In another experiment, 5-day-old tumours developing from a $10^6$ cell inoculum were excised, and the weight determined in untreated ($3.38 \pm 0.27$ g) and piroxicam-treated ($1.25 \pm 0.09$ g) mice. The number of cells recovered from tumours of control mice was $2.0 \times 10^8$ while $1.3 \times 10^8$ cells were recovered from tumours of treated mice. The smaller size of the tumours in treated mice, consequently, does not seem to be related to a decrease in tumoral oedema due to anti-inflammatory treatment. Moreover, incubation of the tumour mass at 60°C until constant weight revealed that the weight loss was 67% in untreated and 83% in treated mice, which indicates that the water relative content was higher in treated than in control mice.

The effects on inflammation of either CI triggered by different irritants or NSAIA treatment were evaluated by

**Table I** Effect of piroxicam on mice survival after s.c. inoculation of LB tumour cells

| *Days* | *Control surviving/total* | *Piroxicam surviving/total* | *P* |
|---|---|---|---|
| 20 | 7/8 | 8/8 | 1 |
| 24 | 6/8 | 8/8 | 0.450 |
| 26 | 1/8 | 8/8 | 0.002 |
| 30 | 1/8 | 7/8 | 0.012 |
| 42 | 0/8 | 2/8 | 0.450 |

**Figure 3** Hind-paw oedema induced by carrageenan injection and cells migrating towards a secondary sponge placed s.c., in mice treated with 0.3 mg $kg^{-1}$ piroxicam, 0.5 mg $kg^{-1}$ indomethcin and mice with a primary s.c. sponge. Control mice were injected i.p. with saline and the group receiving a primary sponge were, in addition, sham-inoculated with a trocar. Since neither paw oedema nor cell migration were affected by this procedure, the results from a single control group are exhibited. Each bar represents the arithmetic mean $\pm$ s.e.m. from eight mice, (*) significant difference (see level of significance in **Results**).

determining (i) the degree of oedema inhibition of the hind-limb footpads and (ii) the inhibition of cell migration towards the site where a second irritative stimulus was delivered. Carrageenan-induced paw oedema was inhibited by an s.c. implanted sponge ($P = 0.029$). A single dose of piroxicam 0.3 mg $kg^{-1}$ or indomethacin 0.5 mg $kg^{-1}$ also inhibited paw oedema ($P = 0.0006$ and $P = 0.0002$, respectively) (Figure 3). Cell migration towards a second s.c. sponge was diminished by treatment with piroxicam ($P = 0.0043$) or indomethacin ($P = 0.0079$). The presence of the first s.c. sponge as the primary irritant decreased the number of cells recovered from the contralateral, second s.c. sponge ($P = 0.0086$) (Figure 3). CI induced by an s.c. injection of $2 \times 10^8$ formalin-killed gram-negative bacteria (*Pseudomonas aeruginosa)* also decreased the number of migrating cells from $(1.5 \pm 0.1) \times 10^6$ (control mice) to $6.5 \times 10^5$ cells $ml^{-1}$ ($P = 0.0086$).

The presence of LB tumour exerted anti-inflammatory activity as measured by decrease of paw oedema and cell

**Figure 4** The effect of an LB solid tumour on oedema induced by carrageenan injection in the hind-footpad (**a**), and on cell migration into the sponge implanted s.c. (**b**). Mice bearing a tumour were evaluated after challenge with $10^6$ and $10^7$ LB cells respectively. Each point represents the arithmetic mean ± s.e.m. from eight mice, (*) significant difference when compared with controls (see text for level of significance). Shaded areas represent the arithmetic mean ± s.e.m of the hind limb weight of (**a**), and the cell number retrieved from (**b**) unchallenged mice.

**Figure 5** Histopathological analysis of skin explants from the area where 1% carrageenan was injected intradermally to control mice (**a**), mice bearing a 7-day-old solid LB tumour (**b**) and mice bearing a 10-day-old solid LB tumour (**c**) (magnification × 2.5). The insets depict, at higher magnification ( × 25) the infiltrated region of the tissue in each case. Representative photomicrographs from groups of six mice are shown.

migration. Paw oedema was diminished on day 4 or 6 after inoculation of $10^6$ LB cells ($P = 0.0069$) when compared with controls (Figure 4a). The number of cells recovered from the sponges were decreased by days 5 and 6 after tumour implantation ($P = 0.029$ and $P = 0.0004$, respectively). On day 7 a rebound effect was observed and the number of cells was increased ($P = 0.029$) (Figure 4b).

The effect of the tumour on cell migration towards an inflammatory i.d. stimulus (carrageenan) was confirmed histologically. In control mice, microscopy of skin explants revealed inflammation characterised by diffuse infiltration involving dermis and subcutaneous fatty tissue (Figure 5a). The infiltrate contained predominantly mononuclear cells (inset of Figure 5a). In contrast, mice bearing a solid LB tumour of 7 days growth exhibited inflammation in a band pattern restricted to a thin layer localised between dermis and fatty subcutaneous tissue (inset Figure 5b). When carrageenan was administered to mice bearing a 10-day tumour, severe inflammation developed, with intense infiltration localised in a band between dermis and fatty subcutaneous tissue, with only diffuse infiltration in these two areas (Figure 5c). At higher magnification, the density of the mononuclear cell infiltrate was even greater than that seen in untreated mice (inset Figure 5c *vs.* inset Figure 5a).

### Discussion

There are two main aspects to be discussed in the light of the results from this study. One relates to CI as a natural mechanism of tumour growth inhibition, and the other is the therapeutic application of NSAIA to obtain inhibition of non-immunogenic tumour growth. Inhibition of inflammation by a secondary irritant stimulus due to CI has been demonstrated in diverse experimental systems. Supporting our results, there is previous evidence suggesting that inflammatory responses may be affected by the presence of a tumour. Snyderman *et al.* (1976) showed that experimental transplantation of a benzopyrene-induced sarcoma in rats prevented Mϕ accumulation during inflammation. Furthermore, Normann *et al.* (1985*a*) have shown that transplantation of syngeneic tumour cells depressed Mϕ inflammatory responses, probably through an increase in the endogenous levels of corticosterone (Normann *et al.*, 1988). Our data demonstrate that, as with immunogenic tumours, a cancer of immunogenicity below our level of detection can also induce CI.

At least two important features of tumour-Mϕ interaction should be considered. The first is that cellular immune responses occurring within a tumour can destroy it (Zbar *et al.*, 1970). In fact, immunologically mediated tumour killing is frequently associated with an influx of Mϕ to the site of neoplastic growth (Shin *et al.*, 1975). On the other hand, migratory cells, especially Mϕ, are required during the early stages of solid tumour growth (Folkman & Cotran, 1976). There were two significant findings in our study: (i) inhibition of tumour growth by an inflammatory focus located away from the site of LB cell grafting; and (ii) inhibition of blood cell migration induced by the LB tumour towards the site where an irritant had been inoculated. Because the LB tumour itself can both induce CI and be affected by anti-inflammation, it would be expected that a primary tumour inoculum could also inhibit the growth of a secondary inoculum of the same tumour.

Inhibition of experimental metastases by a primary tumour has been observed in laboratory animals (Gorelik *et al.*, 1978) and defined as 'concomitant immunity' (Gorelik *et al.*, 1981) or, more appropriately, 'concomitant resistance' (Meiss *et al.*, 1986), a non-specific, rapid, dose-dependent and complex phenomenon. The molecular basis of concomitant resistance remains unknown, but it is speculated that several mechanisms may be acting either simultaneously or sequentially. It has been demonstrated in our laboratory that, although non-immunogenic, the LB tumour can induce concomitant resistance (Ruggiero *et al.*, 1985), and that inhibition of secondary LB tumour growth by the presence of a primary tumour is abrogated if inflammatory Mϕs are injected into the secondary site of tumour cell implantation (Bustuoabad *et al.*, 1984). We detected inhibition of cell migration induced by an LB tumour for a 4–5 day period, coinciding with the early stages of concomitant resistance. If our finding is correct, CI may therefore explain the early inhibition of a secondary inoculum of LB cells; once the non-immunogenic neoplastic tissue is organised and vascularised, secondary tumour growth would not be affected by inhibition of Mϕ migration induced by either CI or treatment with NSAIA.

Administration of different NSAIAs as a single agent to inhibit tumour growth has been attempted (Humes *et al.*, 1974; Lynch *et al.*, 1978; Bennet *et al.*, 1979, 1982, 1985; Narisawa *et al.*, 1981; Panje, 1981; Pollard & Luckert, 1981; McCormick & Moon, 1983; Caignard *et al.*, 1984; Fulton, 1984; Rubio, 1984; McCormick *et al.*, 1985; Kort *et al.*, 1986). Piroxicam treatment inhibits colon carcinogenesis (Pollard & Luckert, 1983; Reddy *et al.*, 1987) in rats, and in combination with an anti-neoplastic agent it has also been used in man (Braun *et al.*, 1987). The conflicting results obtained in these studies may be because of (i) a wide variety of spontaneous and induced tumours used experimentally; (ii) the tumours being transplanted in different sites; (iii) different animal species being used; (iv) NSAIA treatment being started at different times, before or after tumour challenge; and (v) NSAIAs being administered alone or in combination with other drugs (Caignard *et al.*, 1984). The major trend from these studies, however, is that NSAIA can inhibit tumour growth. Because high levels of $PGE_2$ secreted by immunogenic tumours can interfere with immune responses against tumour cells (Normann *et al.*, 1987), it has been postulated that the anti-tumour activity of NSAIAs may be partly due to restoration of immune functions following inhibition of $PGE_2$ synthesis (Lynch *et al.*, 1978; Robertson *et al.*, 1988). Other mechanisms which may explain the anti-tumour activity of NSAIAs include inhibition of blood cell migration, as shown in this study, and the ability of NSAIAs, including piroxicam, to form stable copper complexes (Weser *et al.*, 1982), an important angiogenic factor necessary for neovascularisation (Ziche *et al.*, 1982).

It can be concluded that a syngeneic tumour of non-detected immunogenicity can induce CI, which may play a role in the early stages of concomitant resistance, and that treatment with NSAIA delays LB tumour growth *in vivo*. Further studies ar required to ascertain the usefulness of NSAIA treatment to prevent or delay the appearance of metastases.

The authors thank Dr Christiane Dosne Pasqualini and Dr Anne Morris Hooke for reviewing the manuscript, and Mr Juan Portaluppi for his technical help. The authors also thank Lic. Rafael Repetto and Mr Antonio Morales from our animal house for their help. Piroxicam (Pfizer, Argentina) was obtained by courtesy of Dr Horacio Casabé. This investigation was supported by a grant from Consejo Nacional de Investigaciones Cientificas y Tecnicas, Argentina.

### References

ATKINSON, D.C. & HICKS, R. (1975). The anti-inflammatory activity of irritants. *Agents Actions*, **5**, 239.

ATTIA, M.A.M. & WEISS, D.W. (1966). Immunology of spontaneous mammary carcinomas in mice. V. Acquired tumour resistance and enhancement in strain. A mice infected with mammary tumour virus. *Cancer Res.*, **26**, 1787.

BARRERA, C.N., MAZZOLI, A.B., BUSTUOABAD, O.D., ANDREETA, A.M., & PASQUALINI, C.D. (1985). Macrophages and tumour growth. II. Cell kinetics at the site of allogeneic tumor growth. *Cancer Invest.*, **3**, 7.

BENNET, A., HOUGHTON, J., LEAPER, D.J. & STAMFORD, I.F. (1979). Cancer growth, response to treatment and survival time in mice: beneficial effect of the prostaglandin synthesis inhibitor flurbiprofen. *Prostaglandins*, **17**, 179.

BENNETT, A., BERSTOCK, D.A. & CARROLL, M.A. (1982). Increased survival of cancer-bearing mice treated with inhibitors of prostaglandin synthesis alone or with chemotherapy. *Br. J. Cancer*, **45**, 762.

BENNETT, A., CARROLL, M.A., MELHUISH, P.B. & STAMFORD, I.F. (1985). Treatment of mouse carcinoma in vivo with a prostaglandin $E_2$ analogue and indomethacin. *Br. J. Cancer*, **52**, 245.

BRAUN, D.P., BONOMI, P.D., TAYLOR, S.G., IV & HARRIS, J.E. (1987). Modification of the effects of cytotoxic chemotherapy on the immune responses of cancer patients with a nonsteroidal, anti-inflammatory drug, Piroxicam. A pilot study of the Eastern Cooperative Oncology Group. *J. Biol. Response Mod.*, **6**, 331.

BUSTUOABAD, O.D., GENOVESE, J.A. & PASQUALINI, C.D. (1984). Abrogation of concomitant tumour immunity in mice. *Commun. Biol.* (Buenos Aires), **2**, 423.

CAIGNARD, A., MARTIN, M., REISSER, D., THOMAS, B. & MARTIN, F. (1984). Effects of cimetidine and indomethacin on the growth of dimethylhydrazine-induced or transplanted intestinal cancers in the rat. *Br. J. Cancer*, **50**, 661.

FOLKMAN, J. (1985). Tumour angiogenesis. *Adv. Cancer Res.*, **43**, 175.

FOLKMAN, J. & COTRAN, R. (1976). Relation of vascular proliferation to tumour growth. *Int. Rev. Exp. Pathol.*, **16**, 207.

FULTON, A.M. (1984). *In vivo* effects of indomethacin on the growth of murine mammary tumours. *Cancer Res.*, **44**, 2416.

GOODWIN, J.S. & CEUPPENS, J. (1983). Regulation of the immune response by prostaglandins. *J. Clin. Immunol.*, **3**, 295.

GORELIK, E., SEGAL. S. & FELDMAN, M. (1981). On the mechanism of tumour 'concomitant immunity'. *Int. J. Cancer*, **27**, 847.

HUMES, J.L., CUPO, J.J., JR. & STRAUSSER, H.R. (1974). Effects of indomethacin on Moloney sarcoma virus-induced tumours. *Prostaglandins*, **6**, 463.

KORT, W.J.Z., HULSMAN, L.O.M., VAN SCHALKWIJK, W.P., WEIJMAN, I.M., ZONDERVAN, P.E. & WESTBROEK, D.L. (1986). Reductive effect of aspirin treatment on primary tumour growth and metastasis of implanted fibrosarcoma in rats. *J. Natl Cancer Inst.*, **76**, 711.

LYNCH, N.R., CASTES, M., ASTOIN, M. & SALOMON. J.C. (1978). Mechanism of inhibition of tumour growth by aspiin and indomethacin. *Br. J. Cancer*, **38**, 503.

McCORMICK, D.L. MADIGAN, M.J. & MOON, R.C. (1985). Modulation of rat mammary carcinogenesis by indomethacin. *Cancer Res.*, **45**, 1803.

McCORMICK, D.L. & MOON, R.C. (1983). Inhibition of mammary carcinogenesis by flurbiprofen, a non-steroidal anti-inflammatory agent. *Br. J. Cancer*, **48**, 859.

MEISS, R.P., BONFIL, R.D., RUGGIERO, R.A. & PASQUALINI, C.D. (1986). Histologic aspects of concomitant resistance induced by nonimmunogenic murine tumours. *J. Natl Cancer Inst.*, **76**, 1163.

NARISAWA, T., SATO, M., TANI, M., KUDO, T., TAKAHASHI, T. & GOTO, A. (1981). Inhibition of development of methylnitrosourea-induced rat colon tumours by indomethacin treatment. *Cancer Res.*, **41**, 1954.

NORMANN, S.J., SCHARDT, M. & SORKIN, E. (1985*a*). Antileukocyte activity. I. Systemic inhibition of cellular emigration following local inflammation. *J. Leukocyte Biol.*, **37**, 319.

NORMANN, S.J., SCHARDT, M. & SORKIN, E. (1985*b*). Antileukocyte activity. II. Induction of tolerance to systemic anti-inflammation associated with local irritation and major surgery. *J. Leukocyte Biol.*, **37**, 331.

NORMANN, S.J., SCHARDT, M. & SORKIN, E. (1987). Cancer induced anti-inflammation and its potentiation by tumour excision and rechallenge. *J. Leukocyte Biol.*, **42**, 61.

NORMANN, S.J., BESEDOVSKY, H., SCHARDT, M. & DEL REY, A. (1988). Hormonal changes following tumor transplantation: factors increasing corticosterone and the relationship of corticosterone to tumour-induced anti-inflammation. *Int. J. Cancer*, **41**, 850.

OYANAGUI, Y. (1984). Anti-inflammatory effects of polyamines in serotonin and carrageenan paw edemata – possible mechanism to increase vascular permeability inhibitory protein level which is regulated by glucocorticoids and superoxide radical. *Agents Actions*, **14**, 228.

PANJE, W.R. (1981). Regression of a head and neck carcinoma with a prostaglandin-synthesis inhibitor. *Arch. Otolaryngol.*, **107**, 658.

POLLARD, M. & LUCKERT, P.H. (1981). Effect of indomethacin on intestinal tumours induced in rats by the acetate derivative of dimethylnitrosamine. *Science*, **214**, 558.

POLLARD, M. & LUCKERT, P.H. (1983). The suppressive effect of piroxicam on autochthonous intestinal tumours in the rat. *Cancer Lett.*, **21**, 57.

PREHN, R.T. & PREHN, L.M. (1987). The autoimmune nature of cancer. *Cancer Res.*, **47**, 927.

REDDY, B.S., MARUYAMA, H. & KELLOFF, G. (1987). Dose-related inhibition of colon carcinogenesis by dietry piroxicam, a non-steroidal anti-inflammatory drug, during different stages of rat colon tumour development. *Cancer Res.*, **47**, 5340.

ROBERTSON, B., DOSTAL, K. & DAYNES, R.A. (1988). Neuropeptide regulation of inflammatory and immunologic responses. The capacity of alpha-melanocyte-stimulating hormone to inhibit tumour necrosis factor and IL-1-inducible biologic responses. *J. Immunol.*, **140**, 4300.

RUBIO, C.A. (1984). Antitumoral activity of indomethacin on experimental esophageal tumors. *J, Natl Cancer Inst.*, **72**, 705.

RUGGIERO, R.A., BUSTUOABAD, O.D., BONFIL, R.D., MEISS, R.P. & PASQUALINI, C.D. (1985). 'Concomitant immunity' in murine tumours of non-detectable immunogenicity. *Br. J. Cancer*, **51**, 37.

SHIN, H.S., HAYDEN, M., LANGLEY, S., KALISS, N. & SMITH, M.R. (1975). Antibody-mediated suppresion of grafted lymphoma. III. Evaluation of the role of thymic function, non-thymus-derivated lymphocytes, macrophages, platelets and polymorphonuclear leukocytes in syngeneic and allogeneic hosts. *J. Immunol.*, **114**, 1255.

SNYDERMAN, R., PIKE, M.C., BLAYLOCK, B.L. & WEINSTEIN, P. (1976). Effects of neoplasms on inflammation: depression of macrophage accumulation after tumour implantation. *J. Immunol.*, **116**, 585.

WESER, U., LENGFELDER, E., SELLINGER, K.H. & SCHOBOTZ, L. (1982). Reactivity of chelated copper with superoxide. In *Inflammatory Diseases and Copper*, Sorenson, J.R.J. (ed) p. 513. Humana Press: Clifton, NJ.

ZBAR, B., WEPSIC, H.T., RAPP, H.J., STEWART, L.C. & BORSOS, T. (1970). Two-step mechanism of tumor graft rejection in syngeneic guinea pigs. II. Initiation of reaction by a cell fraction containing lymphocytes and neutrophils. *J. Natl Cancer Inst.*, **44**, 701.

ZICHE, M., JONES, J. & GULLINO, P. (1982). Role of prostaglandin $E_1$ and copper in angiogenesis. *J. Natl Cancer Inst.*, **69**, 475.